# Genomic Exploration of Nonalcoholic Fatty Liver Disease: Insights From Gene Expression and Variation in Morbidly Obese Individuals

**DOI:** 10.1155/jobe/9245699

**Published:** 2025-05-05

**Authors:** Tamadher Abbas Rafaa, Safa Abbas Khudhair, Zahraa Yassen Mohammed, Ahmed AbdulJabbar Suleiman

**Affiliations:** ^1^Department of Higher Education, University Headquarter, University of Anbar, Ramadi, Anbar, Iraq; ^2^Scientific Affairs Department, University Headquarter, University of Anbar, Ramadi, Anbar, Iraq; ^3^Department of Biotechnology, College of Science, University of Anbar, Ramadi, Anbar, Iraq

**Keywords:** cirrhosis, hepatic steatosis, hepatocytes, metabolic syndrome, nonalcoholic fatty liver disease, single nucleotide polymorphism

## Abstract

Nonalcoholic fatty liver disease (NAFLD) is a common liver condition resulting from metabolic syndrome characterized by fat accumulation in the liver. It is often associated with obesity and diabetes, contributing to hepatic steatosis in liver cells. The prevalence of NAFLD is increasing globally, with 32% of the adult population affected. Genetic modifiers, such as single nucleotide polymorphisms, can increase susceptibility to the disease. Gene expression analysis and genetic variation can help identify disease-causing pathways and reveal biomarkers involved in NAFLD. This study employed integrative bioinformatics analysis, including bulk RNA-seq and single-cell RNA-seq, to explore differentially expressed genes and their genetic variants in NAFLD vs. control and NAFLD vs. cirrhosis, highlighting genes influencing NAFLD progression. Moreover, this study identified *AKR1D1, LIPC, UGT2B17, DGAT2,* and *SERPINE1* implicated in metabolic, immune, and lipid functions while being overexpressed in both hepatocyte cells among obese patients identified and validated through Liver Cell Atlas, highlighting their pivotal role in the pathogenesis of the disease in obese patients through perturbed hepatocytes. Furthermore, novel pathogenic variants of *AKR1D1, LIPC,* and *SERPINE1*, associated with congenital bile acid synthesis defects, abnormal circulating lipid concentrations, and plasminogen activator inhibitor type 1 deficiency conditions, were identified. Conclusively, this integrative multiomics study highlights the novel pathogenic variants of *AKR1D1*, *LIPC*, and *SERPINE1* in metabolic, immune, and lipid pathways that are highly expressed among hepatocytes in obese patients while possibly carrying pathogenic mutations that may be associated with NAFLD, emphasizing their potential as novel targets for therapeutic strategies and biomarker development in early diagnosis and treatment before the onset of cirrhosis or hepatocellular carcinoma.

## 1. Introduction

Obesity is linked to a range of liver abnormalities, including nonalcoholic fatty liver disease (NAFLD), a common liver condition that occurs as a result of metabolic syndrome [1]. NAFLD encompasses various liver diseases, including NASH, a severe, advanced condition characterized by inflammation, liver cell damage, and fat accumulation, as well as liver cirrhosis, hepatocellular carcinoma (HCC), cardiovascular disease, and dementia [2, 3].

Recently, the growing prevalence of NAFLD has been characterized by the worsening of liver inflammation and cell death, with or without hepatic fibrosis, which poses a significant burden on public health [4]. NAFLD is often associated with obesity and diabetes, as these metabolic disorders contribute to the pathological accumulation of fat (hepatic steatosis) in over 5% of liver cells (hepatocytes) [5, 6]. The NAFLD prevalence is increasing globally, with 32% of the adult population affected. Individuals with type 2 diabetes (T2D) have the highest age-standardized cumulative mortality rate (41.3%) than those with prediabetes (35.1%) [7]. Moreover, NAFLD risk increases with the severity of obesity. However, only a few obese patients develop NAFLD and advance to cirrhosis [8]. Similarly, the incidence increases with higher body mass index (BMI), with steatosis and steatohepatitis rates around 15% and 3%, respectively [9]. Class I and II obesity rates increase to 65% and 20%, while in extremely obese patients, rates increase to 85% and 40% [10].

Major hepatic metabolic pathways significantly influence liver metabolic homeostasis and systemic health. Dysregulation in these pathways can trigger the pathogenesis of NAFLD, leading to lipotoxicity, impaired lipid metabolism, oxidative stress, and increased endoplasmic reticulum stress (ER stress). These processes subsequently result in the recruitment and activation of inflammatory cells, resulting in liver damage [11]. Similarly, several genetic modifiers have been linked to the progression of NAFLD from steatosis to cirrhosis. The genetic implications of NAFLD are primarily mediated through single nucleotide polymorphisms (SNPs) in genes involved in various aspects of hepatic function, such as fatty acid uptake, lipid droplet biology, transportation of very low-density lipoproteins (VLDLs), de novo lipogenesis (DNL), gluconeogenesis, glycogenolysis, insulin resistance (IR), ER stress, oxidative stress, autophagy, and inflammation [12]. Specific gene variants and their expression levels can significantly influence susceptibility to NAFLD.

A total of 132 genes are reportedly linked with hereditary problems related to hepatic steatosis, with 32 specific locations associated with the development of NAFLD in various human studies [13]. Notably, the hepatic fat content and inflammation are increased by *PNPLA3* and *TM6SF2*, lipid metabolism genes, in all NAFLD disease stages [14]. Additionally, other genes such as *TM6SF2*, *SAMM50*, *PARVB*, *NCAN*, *PPP1R3B*, *GCKR*, and *LYPLAL1* and their variants dysregulate metabolism, immune cell recruitment, and altered signal transduction leading to NAFLD pathogenesis [15, 16]. Furthermore, high expression of immune-related genes, such as interleukins, is reported to cause increased liver inflammation and fibrosis in NAFLD patients [17]. Consequently, gene expression analysis and genetic variation in patients can help identify disease-causing pathways and reveal biomarkers involved in NAFLD [18].

Therefore, an integrative analysis was conducted in this study to identify the NAFLD-dysregulated genes and their pathogenic variants. Bulk RNA sequencing was employed to analyze dysregulated NAFLD gene expressions from datasets compared to cirrhosis and control samples and their implications in metabolic, immune, and lipid functions. Further shortlisting of these genes was done based on their expression in hepatocytes and obese patients. Lastly, the pathogenic variations for shortlisted genes were determined to highlight their potential pathogenic role in disease progression. This approach enhances the understanding of NAFLD-specific genetic markers, their expression, and variants in targeted interventions and personalized treatment strategies. However, experimental investigations must be employed to further validate these findings.

## 2. Methodology

### 2.1. Dataset Selection

The study collected paired-end RNA-sequencing data (ArrayExpress Accession ID: E-MTAB-12807) from the ArrayExpress database (https://www.ebi.ac.uk/arrayexpress). ArrayExpress is a functional genomics data repository used for gene expression and DNA methylation profiling data and serves as the primary data source for Expression Atlas at EMBL-EBI [19]. The data were obtained using the Illumina NovaSeq 6000 platform. The study focused on RNA-seq human liver biopsies from patients. The dataset included a total of nine samples from human liver tissues, with either NAFLD, cirrhosis of the liver, or exhibited normal liver function. Specifically, the dataset includes samples from thirty females and thirty-two males, with ages ranging from 20 years to 79 years. These samples were collected under different physiological conditions, with some obtained postprandially and others during fasting states, providing a comprehensive view of liver tissue characteristics across different metabolic conditions.

### 2.2. RNA-Seq Preprocessing, Mapping, and Postprocessing

The raw reads obtained from the dataset underwent preprocessing to ensure the reliability of subsequent analyses. It is essential to perform this preprocessing to address issues such as low-quality reads, adapter contamination, and primer residues. The FastQC tool (https://www.bioinformatics.babraham.ac.uk/projects/fastqc/) was employed to perform a thorough assessment of the raw reads, evaluating the parameters such as per-base sequence quality, GC content, per-base N content, sequence length distribution, and sequence duplication levels [20]. Subsequently, the Fastp tool (https://github.com/OpenGene/fastp) was used to filter out extraneous elements and decontaminate the raw reads prior to alignment [21]. Following filtering, the cleaned reads were aligned to the reference human genome (GRCh38) using the HISAT2 aligner (https://daehwankimlab.github.io/hisat2/) [22]. The quantification of the aligned reads was performed using the featureCounts tool, which assigns accurate reads to features [23]. Consequently, a gene counts file is created with row headers as gene IDs and column headers as sample IDs.

### 2.3. Differential Gene Expression (DGE) Analysis

DGE analysis was conducted using the DESeq2 package, employing the Wald test [24]. This method estimates gene expression variance and fits a negative binomial distribution to each gene. Furthermore, NAFLD vs. control and NAFLD vs. cirrhosis groups were used for the differential expression of genes by utilizing the quantified read counts. Subsequently, genes that were both statistically and biologically significant were identified from the differentially expressed genes (DEGs) based on criteria such as a *p* value < 0.05, 1 < logFC < −1, and a false discovery rate (FDR) controlled at 5%. This analysis resulted in the identification of upregulated and downregulated genes. Additionally, significant DEGs were visualized using volcano plots.

### 2.4. Pathway Enrichment Analysis of Both NAFLD Groups

The functional pathways in both groups of NAFLD were identified through the Kyoto Encyclopedia of Genes and Genomes (KEGG) by the Enrichr web server (https://maayanlab.cloud/Enrichr/). Enrichr is an extensive resource containing curated gene sets and functions as a search engine that compiles biological knowledge to facilitate further discoveries [25]. Subsequently, the literature review was performed to identify reported KEGG pathways that are associated directly with NAFLD. The upregulated genes implicated in NAFLD-related pathways were identified for both groups.

### 2.5. Identification of Biological Functional Genes From Both Groups

A list of genes was curated for the biological function, including the lipids, immune system, and metabolism retrieved from the Gene Ontology Resource project (https://geneontology.org/) and comprehensive literature review. Additionally, the immune system genes were also retrieved from the ScType database (https://sctype.app/database.php). The Gene Ontology (GO) provides extensive information on gene functions, accessible in human and machine-readable formats, enabling computational analysis for genetics experiments in biomedical research [26]. ScType is a tool designed to automate the identification of cell types from single-cell RNA-seq data [27]. The NAFLD-related upregulated pathway genes from both groups were then searched against this curated list to determine their biological function.

### 2.6. Identifying Biological Functional Hub Genes in Hepatocytes and Obese Patients

Subsequently, protein–protein interaction (PPI) analysis was performed on the identified biological functional genes in both groups through STRING (https://string-db.org/), a comprehensive database compiling PPIs from other databases, literature, and experimental validations [28]. The networks were visualized with Cytoscape (https://cytoscape.org/), a well-known platform for exploring biological networks [29]. CytoHubba within Cytoscape identified the top 5 hub genes in the PPI networks for both groups.

Lastly, the expression of the top 5 hub genes that demonstrated biological functions related to lipids, the immune system, or metabolism was validated in hepatocytes and obese patients through the Human Liver Atlas (https://www.livercellatlas.org/), a liver single-cell RNA-seq database that provides detailed expression profile of genes or proteins [30]. The genes of both groups, identified in NAFLD-related pathways, reported in biological functions, and expressed in hepatocytes of obese patients, were shortlisted for further analysis.

### 2.7. Retrieval of Pathogenic Variants of Shortlisted Genes

The pathogenic variants of the shortlisted genes for NAFLD were retrieved through the ClinVar (https://www.ncbi.nlm.nih.gov/clinvar/) database. ClinVar is an openly accessible public repository that catalogs reports on human variations categorized by their associations with diseases and drug responses, supported by evidence [31].

## 3. Results

### 3.1. Identification of DEGs

**Figure 1:**
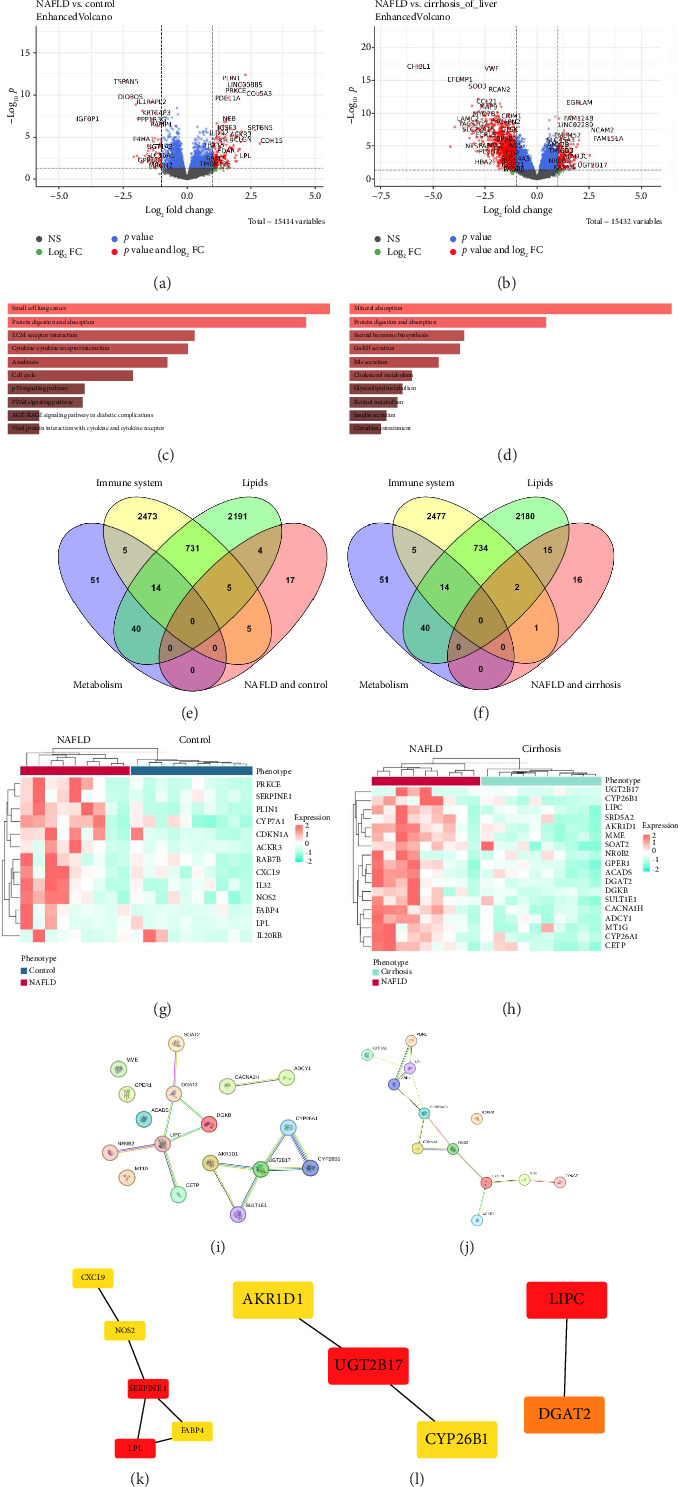
Comprehensive transcriptomic analysis comparing NAFLD vs. control and NAFLD vs. cirrhosis groups. (a-b) Volcano plots displaying significantly dysregulated genes (upregulated and downregulated) between NAFLD vs. control and NAFLD vs. cirrhosis groups, respectively. (c-d) KEGG pathway enrichment analysis showing the top 10 upregulated pathways associated with differentially expressed genes in NAFLD vs. control and NAFLD vs. cirrhosis groups, respectively. (e-f) Venn diagrams identifying common biological functional genes between NAFLD vs. control and NAFLD vs. cirrhosis comparisons, respectively. (g-h) Heatmaps depicting the expression patterns of significantly upregulated and downregulated genes in NAFLD vs. control and NAFLD vs. cirrhosis groups, respectively. (i-j) STRING-based protein–protein interaction (PPI) networks illustrating the interactions among biological functional genes in NAFLD vs. control and NAFLD vs. cirrhosis groups, respectively. (k-l) Identification of key hub genes with high connectivity from the PPI networks in NAFLD vs. control and NAFLD vs. cirrhosis groups, respectively.

The significant DEGs were identified in both groups (NAFLD vs. control and NAFLD vs. cirrhosis) using the DESeq2 package. The comparison between the NAFLD and control groups revealed a total of 179 genes that showed dysregulation, with 135 genes showing upregulation and 44 genes exhibiting downregulation (Figure 1(a)). Similarly, the NAFLD and cirrhosis group identified 1033 significantly dysregulated genes, with 142 upregulated genes, while 891 genes were found to be downregulated (Figure 1(b)).

### 3.2. Pathway Enrichment Analysis of Both NAFLD Groups

The functional enrichment analysis of upregulated genes of both groups was performed to highlight their roles in promoting lipid accumulation, inflammation, and impaired metabolic regulation associated with NAFLD. These upregulated NAFLD vs. control group genes were implicated in various significant NAFLD-related KEGG pathways, such as protein digestion and absorption, extracellular matrix (ECM)–receptor interaction, amebiasis, p53 signaling pathway, cell cycle, peroxisome proliferator–activated receptor (PPAR) signaling pathway, cytokine-cytokine receptor interaction, and AGE-RAGE signaling pathway in diabetic complications (Figure 1(c)). Similarly, the upregulated NAFLD vs. cirrhosis group genes were implicated in mineral absorption, protein digestion and absorption, steroid hormone biosynthesis, GnRH secretion, bile secretion, cholesterol metabolism, glycerolipid metabolism, retinol metabolism, beta-alanine metabolism, and galactose metabolism pathways (Figure 1(d)). Notably, the protein digestion and absorption pathway was common in both groups. The association of these pathways with NAFLD was further validated through an extensive literature review. A total of 31 genes were identified for the NAFLD vs. control group and 34 genes in the NAFLD vs. cirrhosis group. The NAFLD-related pathways and their respective genes from both groups are listed in Table 1.

### 3.3. Identification of Biological Functional Genes From Both Groups

Genes associated with lipid metabolism, the immune system, and metabolism were identified through the GO database, the ScType database, and an extensive literature review. A total of 2985 genes were retrieved for lipids, 3233 genes for the immune system, and 110 genes for metabolism. These identified genes were curated in one list and searched against the NAFLD-related upregulated pathway genes from both the NAFLD vs. control and NAFLD vs. cirrhosis to determine their specific biological functions. A total of 18 biological functional genes were identified for NAFLD vs. cirrhosis and 15 for NAFLD vs. control.

Notably, 17 lipid associated genes (*CYP26A1, UGT2B17, SOAT2, CACNA1H, MME, LIPC, CETP, ACADS, SRD5A2, SULT1E1, AKR1D1, ADCY1, NR0B2, DGAT2, GPER1, CYP26B1,* and *DGKB*) and 3 immune system associated genes (*MT1G, CYP26B1,* and *GPER1*) were found in NAFLD vs. cirrhosis group. Among these, *CYP26B1* and *GPER1* were found to be common genes involved in both lipid and the immune system. Subsequently, 9 genes associated with lipid (*PLIN1, SERPINE1, LPL, FABP4, CYP7A1, NOS2, CDKN1A, PRKCE,* and *CXCL9*) and 10 genes related to the immune system (*IL32, IL20RB, PRKCE, NOS2, RAB7B, CXCL9, ACKR3, LIF, SERPINE1,* and *CDKN1A*) were identified in NAFLD vs. control group. Notably, *SERPINE1, NOS2, CDKN1A, PRKCE,* and *CXCL9* were found to be common genes involved in both lipid and the immune system in the aforementioned group. However, no gene associated with metabolism was found in both groups. The biological functional genes identified in both groups are illustrated through the Venn diagram in Figures 1(e) and 1(f). The expression of the biological functional genes in both groups was visualized through a heatmap (Figures 1(g) and 1(h)).

### 3.4. Identifying Biological Functional Hub Genes in Hepatocytes and Obese Patients

Subsequently, STRING analysis was performed on the biological functional genes for both groups to identify PPIs. It was observed that *SOAT2, DGAT2, DGKB, LIPC, CETP, NR0B2, CACNA1H, ADCY1, AKR1D1, SULT1E1, UGT2B17, CYP26A1,* and *CYP26B1* showed interactions in NAFLD vs. cirrhosis group (Figure 1(i)). However, all the genes except IL20RB showed interactions in NAFLD vs. control (Figure 1(j)).

Moreover, the hub genes were identified by utilizing the STRING database output, which revealed the top 5 hub genes for both groups. Among all, the top 5 hub genes identified for NAFLD vs. control were *CXCL9, NOS2, SERPINE1, FABP4,* and *LPL* (Figure 1(k)), whereas *AKR1D1, UGT2B17, CYP26B1*, *LIPC,* and *DGAT2* were identified as the top 5 hub genes for the NAFLD vs. cirrhosis group (Figure 1(l)). Furthermore, the expression of these hub genes was validated in hepatocytes and obese patients through the Human Liver Atlas. Among all, only *AKR1D1, LIPC, UGT2B17, DGAT2,* and *SERPINE1* hub genes showed expression in hepatocytes and obese patients. Consequently, these hub genes were then shortlisted for further analysis.

### 3.5. Retrieval of Pathogenic Variants of Shortlisted Genes

**Figure 2:**
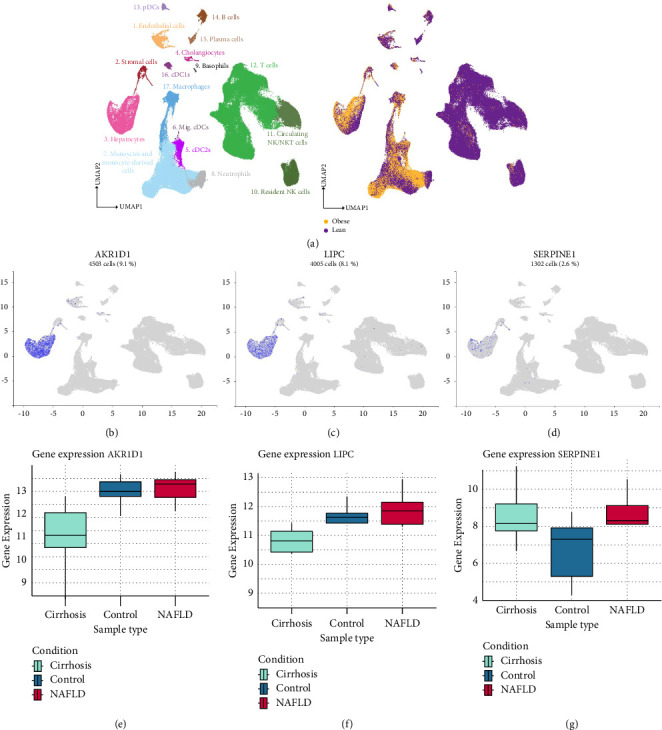
Umap and boxplot of shortlisted genes (a–d) umap of *AKR1D1, LIPC,* and *SERPINE1* genes in hepatocytes and obese cells (e–g) boxplot of *AKR1D1, LIPC,* and *SERPINE1* genes in both conditions.

The pathogenic variants related to NAFLD of the shortlisted genes were identified through ClinVar. Notably, only *AKR1D1, LIPC,* and *SERPINE1* showed NAFLD-related pathogenic variants in ClinVar. Among these genes, *AKR1D1* showed the highest pathogenic mutations, with 12 variants, whereas *LIPC* showed 4 pathogenic mutations. However, *SERPINE1* showed only 2 pathogenic variants. The pathogenic mutations of the aforementioned hub genes are provided in Supporting Information (a), (b), and (c). Lastly, the expression of these genes with pathogenic variants was visualized in both groups, as well as in hepatocytes and obese cells (Figure 2).

## 4. Discussion

NAFLD is a common liver disease associated with obesity, metabolic syndrome, and systemic diseases like cardiovascular disease and dementia. Its genetic mechanisms involve numerous genes and variants related to lipid metabolism, immune response, and metabolic processes. Dysregulation and pathogenic variants contribute to disease progression, necessitating detailed genetic analysis [11]. Therefore, this study utilized integrative analysis, including bulk RNA-seq and single-cell RNA-seq, to explore the molecular and functional aspects of DEGs and their pathogenic variants in NAFLD among obese patients.

Notably, the protein digestion and absorption pathway was dysregulated in both groups, responsible for influencing liver zonation and IR [32]. The NAFLD vs. cirrhosis analysis also showed multiple upregulated pathways, including mineral absorption, steroid hormone–induced lipid homeostasis, and GnRH secretion, that are reported to be responsible for the high prevalence of NAFLD [33–35]. Additionally, the upregulation of the bile-salt export pump (BSEP) and glycerolipid and hepatic cholesterol metabolism pathways causes insulin signaling impairment and atherosclerosis that leads to disease severity [36]. Similarly, serum retinol levels and galactose consumption are associated with inflammation and liver damage [37, 38]. Lastly, the beta-alanine metabolism pathway is also reported as the potential target in NAFLD [39].

In the NAFLD vs. control group, significant dysregulation was observed in numerous pathways, such as ECM receptor interactions, higher amebiasis prevalence, and the complex role of p53 in disease progression [40–42]. Similarly, the cell cycle and the critical role of PPAR pathways also showed dysregulation [43, 44]. Lastly, the cytokine-cytokine receptor interactions and the AGE-RAGE signaling pathway, which cause inflammation and fibrosis, also cause NAFLD progression [17].

Numerous gene studies suggest multiple-hit hypotheses involving genes involved in hepatic lipid metabolism, insulin signaling, and oxidative stress and inflammation, potentially contributing to NAFLD presence and progression [45]. Consequently, the genes from the NAFLD-related pathways from both groups, associated with lipid metabolism, the immune system, and metabolism, were identified among the NAFLD-related pathway genes.

Additionally, the top 5 biological functional hub genes in NAFLD vs. control (*CXCL9, NOS2, SERPINE1, FABP4,* and *LPL*) and NAFLD vs. cirrhosis (*AKR1D1, UGT2B17, CYP26B1*, *LIPC,* and *DGAT2*) groups were identified through PPI analysis among lipid, immune, and metabolism dysregulated genes. Notably, all hub genes identified in the NAFLD vs. control groups are found to be upregulated, contributing to the development and progression of NAFLD. Similarly, most hub genes in the NAFLD vs. cirrhosis group were dysregulated, except for *UGT2B17* and *CYP26B1*. While the dysregulation of *UGT2B17* and *CYP26B1* genes is not directly associated with NAFLD, their role in metabolic processes leading to the disease is reported [46, 47].

NAFLD is strongly associated with obesity, which is a characteristic of clinically suspected fatty liver. Obesity-associated hepatic tumorigenesis develops from NAFLD, progressing to NASH, cirrhosis, and ultimately HCC. Around 50% of NAFLD patients and 80% of NASH patients present with obesity [48]. Furthermore, NAFLD develops due to a disruption in lipid metabolism in the hepatocytes, leading to the buildup of triglyceride-rich droplets in the liver. This imbalance in fatty acid uptake and synthesis initiates a cascade of adverse effects, including lipotoxicity, oxidative stress, inflammation, and fibrosis, which collectively contribute to severe liver damage. The fat mass and obesity-associated (FTO) gene, a susceptibility gene for obesity, is linked to the severity of NAFLD, exhibiting high expression in fat and liver tissues [49]. Therefore, the expression of these hub genes was validated in hepatocytes and obese cells through the Human Liver Atlas. Notably, only *AKR1D1, LIPC, UGT2B17, DGAT2,* and *SERPINE1* hub genes showed expression in hepatocytes and obese cells. Similarly, it has been reported that gene variants are linked to metabolic disorders, including central obesity, LDL, IR, and hypertriglyceridemia, which are closely linked to NAFLD, suggesting significant implications [50]. Thus, the pathogenic variants of the hub genes from both groups, which showed expression in hepatocytes and obese cells, were retrieved through ClinVar. The *AKR1D1* and *LIPC* genes exhibited variants primarily in congenital bile acid synthesis defect 2 condition. In addition, the *LIPC* gene also showed mutations in the abnormal circulating lipid concentration condition. Lastly, the variants of the *SERPINE1* gene showed mutations in the transcription level of plasminogen activator inhibitor 1 and congenital plasminogen activator inhibitor type 1 deficiency conditions. All of these conditions are reported to be related to NAFLD [51–54].

In summary, this study elucidates the novel identification of pathogenic variants and their biological functions implicated in NAFLD pathogenesis. Notably, *AKR1D1*, *LIPC*, and *SERPINE1* genes showed implications in metabolic, immune, and lipid functions and significant expression in both hepatocytes and obese cells. The pathogenic variants of *AKR1D1* and *LIPC* genes are primarily associated with congenital bile acid synthesis conditions. Moreover, the *LIPC* gene also showed mutations to abnormal circulating lipid concentration conditions. Lastly, the *SERPINE1* variants were associated with the transcription level of plasminogen activator inhibitor 1 and congenital plasminogen activator inhibitor type 1 conditions. These findings suggest that *AKR1D1*, *LIPC*, and *SERPINE1* genes can be considered novel biomarkers and therapeutic targets for NAFLD.

## 5. Conclusion

This study pinpointed dysregulated genes and their variants in NAFLD, revealing implicated biological functions, pathways, and key hub genes (AKR1D1, LIPC, UGT2B17, DGAT2, and SERPINE1) linked to metabolic, immune, and lipid processes in hepatocytes and obese cells. Novel pathogenic variants in AKR1D1, LIPC, and SERPINE1 were found. AKR1D1 and LIPC relate to bile acid synthesis defects and abnormal lipid levels, respectively. SERPINE1 variants show transcription changes in plasminogen activator inhibitor conditions. These genetic insights are significant for NAFLD understanding and suggest potential therapeutic targets and diagnostic biomarkers, pending experimental validation.

## Figures and Tables

**Table 1:** NAFLD-related KEGG pathways and their respective genes.

Pathway	Genes
*NAFLD vs. control group*	
Protein digestion and absorption	ACE2, SLC6A19, MEP1B, COL5A3, COL4A3, COL4A5
ECM-receptor interaction	LAMC3, COL4A3, COL4A5, NPNT, FREM2
Cytokine-cytokine receptor interaction	IL32, EDAR, CXCL9, CCL3L3, LIF, IL20RB, ACKR3
Amebiasis	NOS2, LAMC3, COL4A3, COL4A5, RAB7B
Cell cycle	CDKN1A, CDC45, ESPL1, E2F1, CDC6
p53 signaling pathway	CDKN1A, RRM2, TP53I3, SERPINE1
PPAR signaling pathway	FABP4, LPL, PLIN1, CYP7A1
AGE-RAGE signaling pathway in diabetic complications	PRKCE, SERPINE1, COL4A3, COL4A5

*NAFLD vs. cirrhosis group*	
Mineral absorption	MT2A, MT1A, MT1M, MT1F, MT1G, MT1X, MT1E
Protein digestion and absorption	KCNK5, COL28A1, MME, COL25A1, COL11A2, SLC16A10
Steroid hormone biosynthesis	SULT1E1, SRD5A2, AKR1D1, UGT2B17
GnRH secretion	GPER1, KCNN2, CACNA1H, KCNJ3
Bile secretion	UGT2B17, KCNN2, ADCY1, NR0B2
Cholesterol metabolism	CETP, LIPC, SOAT2
Glycerolipid metabolism	LIPC, DGAT2, DGKB
Retinol metabolism	CYP26A1, CYP26B1, UGT2B17
Insulin secretion	KCNN2, ADCY1, GCK
Circadian entrainment	ADCY1, CACNA1H, KCNJ3

## Data Availability

All data generated or analyzed during this study are included in this published article and its supporting information.

## Supporting Information

The supporting document presents details of pathogenic mutations in three genes—AKR1D1, LIPC, and SERPINE1. For each gene, the file includes the associated SNP ID (single nucleotide polymorphism identifier) and the germline classification. These genes are involved in key physiological processes such as bile acid synthesis (AKR1D1), lipid metabolism (LIPC), and regulation of blood clot breakdown (SERPINE1), highlighting their clinical significance in inherited disorders.
