# Supplementary material for: Genomic Exploration of Nonalcoholic Fatty Liver Disease: Insights From Gene Expression and Variation in Morbidly Obese Individuals
Source: J Obes. 2025 May 5;2025:9245699. doi: 10.1155/jobe/9245699 (PMC12069845; doi:10.1155/jobe/9245699)
Supplement: Supporting Information — Additional supporting information can be found online in the Supporting Information section. [file 9245699.f1.docx]

**a: Pathogenic mutation in AKR1D1 (Aldo-keto reductase family 1 member D1, Bile acid synthesis enzyme) gene with SNP ID and germ line classification**

**b: Pathogenic mutation in LIPC (Lipase C, Hepatic Type, Breaks down fats in blood) gene with SNP ID and germ line classification**

**c: Pathogenic mutation in SERPINE1 (Serpin Family E Member 1, Inhibits enzymes that break down clots) gene with SNP ID and germ line classification**
